# The fear of COVID-19 contagion: an exploratory EEG-fMRI study

**DOI:** 10.1038/s41598-024-56014-4

**Published:** 2024-03-04

**Authors:** Giovanni Federico, Giuseppina Ciccarelli, Giuseppe Noce, Carlo Cavaliere, Ciro Rosario Ilardi, Liberatore Tramontano, Vincenzo Alfano, Giulia Mele, Angelica Di Cecca, Marco Salvatore, Maria Antonella Brandimonte

**Affiliations:** 1 IRCCS SYNLAB SDN, Naples, Italy; 2 Laboratory of Experimental Psychology, Suor Orsola Benincasa University, Naples, Italy

**Keywords:** Cognitive neuroscience, Human behaviour, Public health

## Abstract

Pandemics have the potential to change how people behave and feel. The COVID-19 pandemic is no exception; thus, it may serve as a "challenging context" for understanding how pandemics affect people's minds. In this study, we used high-density electroencephalography (EEG) and functional magnetic resonance imaging (fMRI) to examine the neural correlates of fear of contagion during the most critical moments of COVID-19 in Italy (i.e., October 2020–May 2021). To do that, we stimulated participants (N = 17; nine females) with artificial-intelligence-generated faces of people presented as healthy, recovered from COVID-19, or infected by SARS-CoV-2. The fMRI results documented a modulation of large bilateral fronto-temporo-parietal functional brain networks. Critically, we found selective recruitment of cortical (e.g., frontal lobes) and subcortical fear-related structures (e.g., amygdala and putamen) of the so-called social brain network when participants observed COVID-19-related faces. Consistently, EEG results showed distinct patterns of brain activity selectively associated with infected and recovered faces (e.g., delta and gamma rhythm). Together, these results highlight how pandemic contexts may reverberate in the human brain, thus influencing most basic social and cognitive functioning. This may explain the emergence of a cluster of psychopathologies during and after the COVID-19 pandemic. Therefore, this study underscores the need for prompt interventions to address pandemics' short- and long-term consequences on mental health.

## Introduction

How does the human brain deal with a pandemic? Nowadays, such a question represents an epistemological priority of the cognitive science agenda. Indeed, over the past four years, billions of people from all over the world confronted themselves with an unprecedented event: the first airborne beta-coronavirus (i.e., SARS-CoV-2) becoming a global concern^1^. While unique in its biological characterisation, the COVID-19 pandemic had several psychosocial repercussions that appear to be shared by all other pandemics that have plagued humanity over the centuries^2^. Ultimately, one may reasonably imagine that, under the threat of potentially lethal diseases and due to the pandemic-related upheavals of social habits, individuals may feel, think, and act differently than under non-pandemic conditions^3–5^. However, unlike in past pandemics, modern research can use advanced technologies, such as neuroimaging, to scrutinise the impact of pandemics on the human brain^6^. Therefore, the COVID-19 pandemic may be considered an ecological setting to study individuals' cognitive and social functioning under uncertainty and risk contexts.

Pandemics are stressful events. Indeed, during the most intense moments of a viral pandemic, people may develop a specific form of stress constantly fed by new waves of viruses as well as new variants^7^. Unsurprisingly, a crucial precursor of such a type of stress is the fear of contagion. People are scared about the possibility of coming into contact with viruses, living intense experiences of fear that, in some cases, require specific psychological interventions^8^. Fear is a most basic and essential emotion for the survival of individuals and may be elicited by the perception of dangerous stimuli, namely when people encounter significant threats to their safety^9,10^. Such emotion involves a complex interplay of brain networks as neural correlates, including the limbic system and other subcortical and cortical regions, with a major engagement of the frontal lobes for its regulation^11–13^. Predictably, the fear of contagion is closely associated with airborne viruses such as SARS-CoV-2, as the possibility of infection through interpersonal encounters is high^14^. This fear can impact cognitive and social functioning^15^. Importantly, the stress related to the fear of an invisible threat, namely, the virus, can be linked to the emergence of various mental health issues during the COVID-19 pandemic^2^. However, the neural mechanisms underlying the changes in behaviour, emotion, and cognition related to the pandemic remain largely unexplored. This might be due to the limited opportunities for conducting neuroscientific research during a pandemic crisis. Nonetheless, understanding how pandemic contexts can affect the brain while individuals are experiencing them, particularly during an ongoing event like the COVID-19 pandemic, could offer valuable insights for clinical interventions and help in addressing the long-term consequences of pandemics on mental health^16,17^.

**Figure 1:**
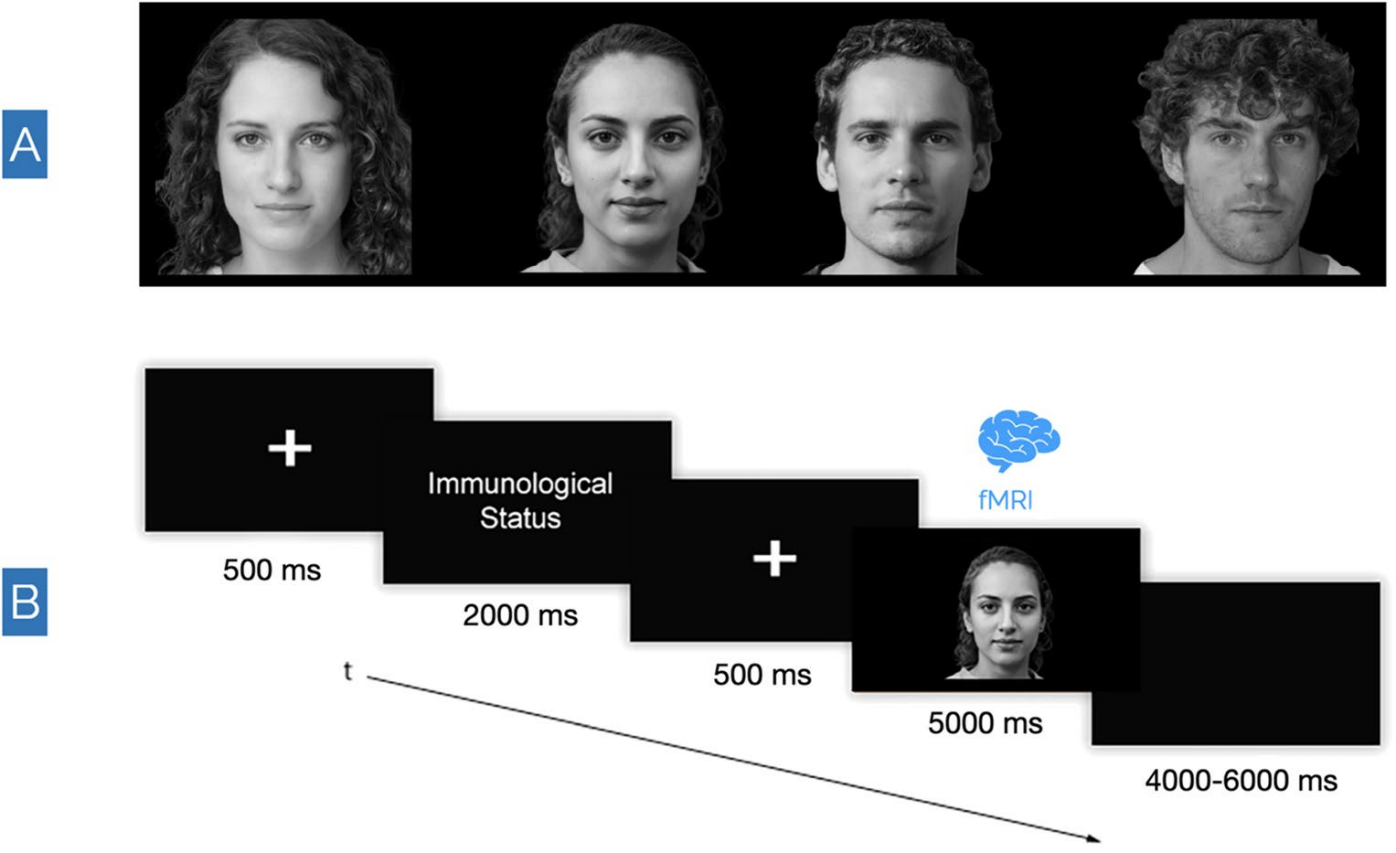
Stimuli and experimental flow. (**A**) Example of artificial-intelligence-generated faces used in the experiment. These images were generated through machine learning by implementing a generative adversarial network^18^. (**B**) The experimental visual flow. For 500 ms, a fixation point appeared, followed by a label indicating the Immunological Status of the subsequent face (i.e., Healthy, Recovered, or Infected), which appeared for 2 s. Then a second fixation point (500 ms) appeared, followed by a face stimulus that lasted 5 s on the screen. Finally, a black screen appeared for 4–6 s.

In this exploratory study, which we devised and carried out during the most critical moments of COVID-19 pandemic in Italy (i.e., October 2020–May 2021), we implemented high-density electroencephalography (EEG) and functional magnetic resonance imaging (fMRI) to study the impact of a pandemic context on the human brain. Specifically, we employed most of the original procedures devised by Federico and colleagues to investigate how people may change their social and cognitive functioning depending on the fear of SARS-CoV-2 infection^15^. In particular, to manipulate the degree of the perceived risk of contagion, we stimulated participants with artificial-intelligence-generated^18^ faces of individuals that were randomly presented as healthy (i.e., stimuli producing no perceived risk of contagion), infected by SARS-CoV-2 (i.e., stimuli plausibly prompting higher perceived risk of contagion) or recovered from COVID-19 (i.e., stimuli characterised by an uncertain level of risk) (Fig. 1). We predicted to differentiate EEG regional spectral activity and MRI functional connectivity as a function of the experimental conditions. In particular, we hypothesised selective recruitment of cortical and subcortical brain regions associated with fear and risk perception^9,19–22^ for COVID-19-related faces compared to healthy ones^15^.

## Results

### fMRI results

**Figure 2:**
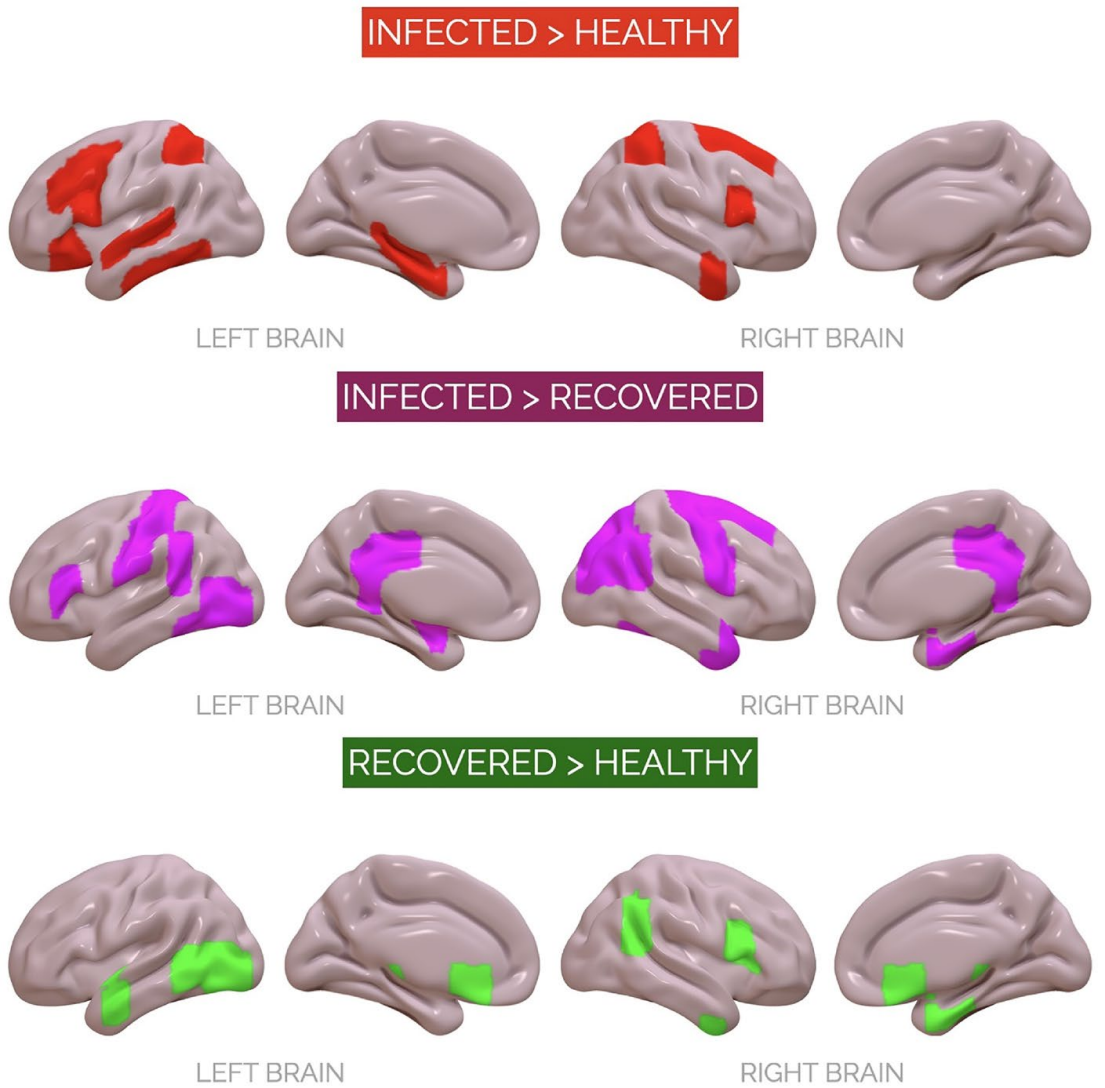
Functional brain networks elicited by experimental manipulations. From top to bottom: ROIs involved in the functional networks related to the contrast between (1) infected and healthy faces, in red; (2) infected and recovered faces, in violet; (3) recovered vs. healthy faces, in green. See Table 1 for ROI-to-ROI Functional Connectivity values and statistics.

fMRI results exhibited a pattern of co-activations related to a large bilateral functional brain network, with selective involvement of specific cortical and subcortical regions depending on the experimental condition. Frontal (e.g., superior and inferior frontal gyrus and orbitofrontal cortex), limbic (e.g., putamen and amygdala), and temporo-mesial (e.g., hippocampus) co-activations seemed to characterise higher-risk conditions (i.e., infected faces) exclusively. Also, the involvement of both sides of the ventral occipitotemporal regions of the brain (such as the inferior and medial temporal gyrus, lateral occipital cortex, and parahippocampal gyrus) was observed across all the experimental conditions. No gender effects emerged. Results are summarised in Table 1 and Fig. 2.

**Table 1:** ROI-to-ROI functional connectivity analysis.

Seed	Target	Beta	T(16)	*P*
Infected Faces > Healthy Faces				
Left MidFG	Left FOrb	0.11	5.09	< .05
Right SFG	Left aSTG	0.13	5.30	< .01
Left IFG op	Right SPL	0.09	5.10	< .05
Right aITG	Left aPaHC	− 0.14	− 6.35	< .01
Left pITG	Right aMTG	0.13	5.28	< .05
Left toITG	Left ICC	0.13	4.94	< .05
Left SPL	Left pITG	− 0.10	− 5.07	< .05
Left aPaHC	Right aITG	− 0.14	− 5.53	< .01
Left aPaHC	Right IFG op	0.11	5.11	< .01
Left Hippocampus	Left pSTG	0.10	5.49	< .01
Infected Faces > Recovered Faces				
Right SFG	Left PostCG	0.12	5.14	< .05
Left IFG tri	Putamen	− 0.12	− 4.86	< .05
Left Amygdala	Right PreCG	0.15	4.85	< .05
Left toITG	Left pSMG	0.13	5.26	< .05
Left PostCG	Right SFG	− 0.11	− 4.54	< .05
Left PostCG	Right AG	0.13	4.42	< .05
Right sLOC	Right TP	− 0.08	− 5.16	< .05
Left iLOC	Right AG	0.09	5.59	< .01
PCC	Right TOFusC	0.13	5.21	< .05
Right aPaHC	Right aITG	0.13	5.48	< .01
Recovered Faces > Healthy Faces				
Left aMTG	Brainstem	− 0.12	− 4.94	< .05
Left toMTG	Right IFG op	0.11	5.32	< .01
Left iLOC	Left PP	0.11	4.79	< .05
SubCalC	Right aITG	− 0.09	− 5.46	< .01
Right aPaHC	Right aITG	− 0.13	− 5.06	< .05
Right FO	Right AG	− 0.14	− 4.77	< .05

MidFG = Middle Frontal Gyrus; FOrb = Frontal Orbital Cortex; IFG op = Inferior Frontal Gyrus, *pars opercularis*; IFG tri = Inferior Frontal Gyrus, *pars triangularis*; SPL = Superior Parietal Lobule; aITG = Inferior Temporal Gyrus, anterior division; aPaHC = Parahippocampal Gyrus, anterior division; aMTG = Middle Temporal Gyrus, anterior division; pITG = Inferior Temporal Gyrus, posterior division; toITG = Inferior Temporal Gyrus, temporooccipital part; ICC = Intracalcarine Cortex; toMTG = Middle Temporal Gyrus, temporooccipital part; iLOC = Lateral Occipital Cortex, inferior division; PP = Planum Polare Left; SubCalC = Subcallosal Cortex; FO = Frontal Operculum Cortex; AG = Angular Gyrus; PreCG = Precentral Gyrus; PostCG = Postcentral Gyrus; SFG = Superior Frontal Gyrus; sLOC = Lateral Occipital Cortex, superior division; TP = Temporal Pole; PCC = Posterior Cingulate Cortex; TOFusC = Temporal Occipital Fusiform Cortex; pSMG = posterior division of the supramarginal gyrus; P = p-value with False Discovery Rate correction.

### EEG results

**Figure 3:**
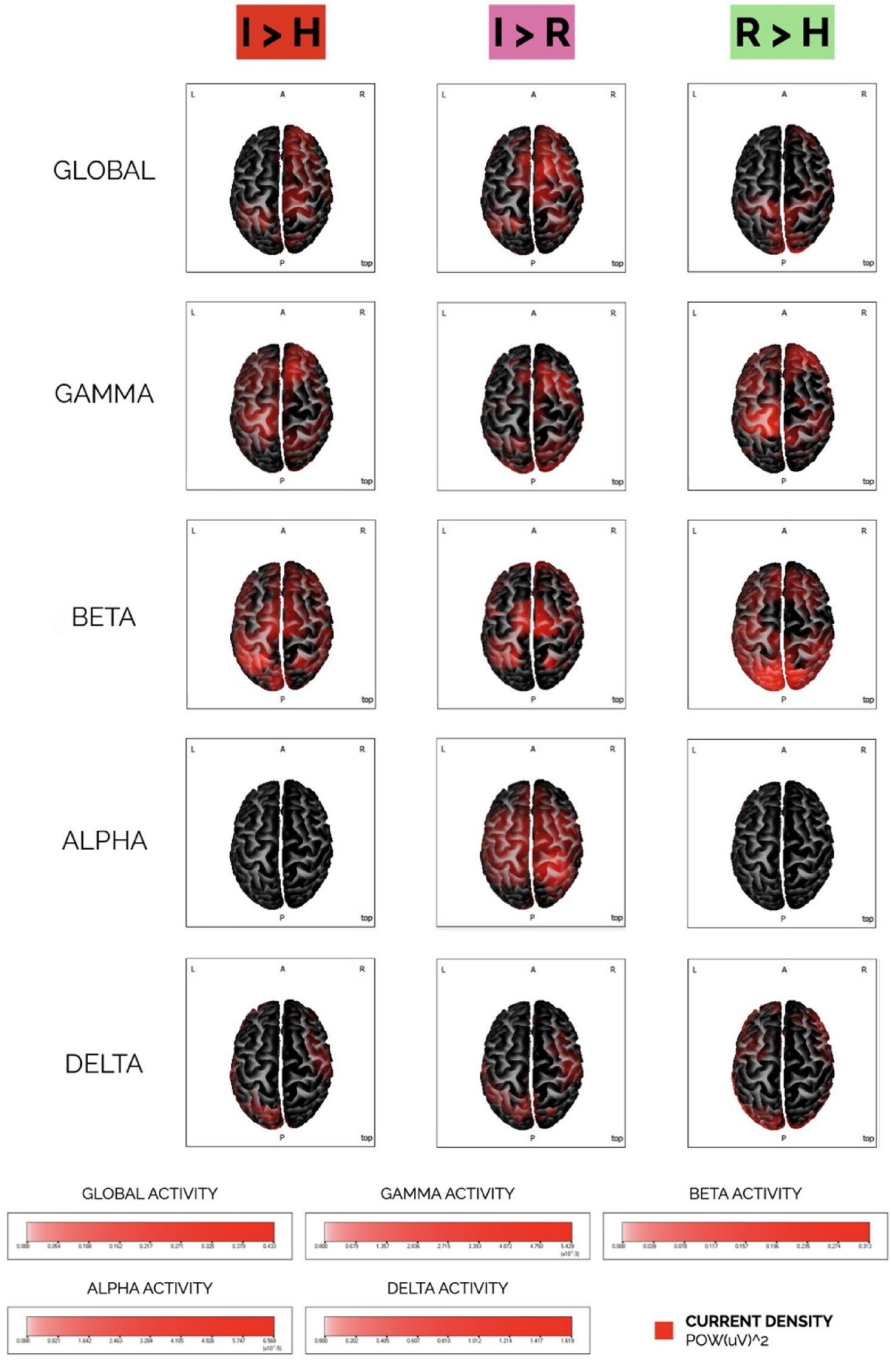
EEG qualitative source analysis (eLORETA). Global and band-specific EEG spectral activity (*i.e.*, current density) for gamma, beta, alpha, and delta frequencies under varying experimental conditions. I = infected faces; H = healthy faces; R = recovered faces.

Data from EEG exact low-resolution brain electromagnetic tomography (eLORETA)^23^ revealed specific involvement of bilateral fronto-temporo-parietal and occipito-parietal brain networks, modulated by experimental conditions. We computed a grand mean of eLORETA current densities across a frequency range of 2–44 Hz, followed by frequency-specific eLORETA solutions: gamma (30.5–44 Hz), beta (13–30 Hz), alpha (8–13 Hz), and delta (2–4 Hz). These EEG eLORETA results, summarized in Fig. 3, showed cortical source activations that overlapped with co-activation patterns observed in MRI functional connectivity analysis. In the time–frequency spectral EEG analysis, Infected Faces elicited higher alpha and gamma activities compared to Healthy Faces. Specifically, Infected Faces exhibited increased alpha activity in the occipital region of the left brain [t(16) = 1.76; *p* < 0.05] and heightened gamma activity across multiple left brain regions—frontal [t(16) = 1.98; *p* < 0.05], central [t(16) = 2.12; *p* < 0.05], parietal [t(16) = 2.51; *p* < 0.05], occipital [t(16) = 2.53; *p* < 0.05], and temporal [t(16) = 2.04; *p* < 0.05]. In the right brain, increased gamma activity in Infected Faces was observed in the central [t(16) = 2.07; *p* < 0.05], parietal [t(16) = 2.26; *p* < 0.05], and occipital [t(16) = 2.58; *p* < 0.05] regions. Furthermore, Infected Faces showed higher alpha and delta activities than Recovered Faces. In the right brain's parietal [t(16) = 2.44; *p* < 0.05] and occipital [t(16) = 2.45; *p* < 0.05] regions, alpha activity was greater in Infected Faces. Delta activity was also higher in Infected Faces, both in the right brain's frontal [t(16) = 1.87; *p* < 0.05], central [t(16) = 1.8; *p* < 0.05], parietal [t(16) = 2.55; *p* < 0.05], and occipital [t(16) = 2.73; *p* < 0.05] regions, and in the left brain's parietal [t(16) = 1.78; *p* < 0.05] and occipital [t(16) = 1.85; p < 0.05] regions. Recovered Faces, compared to Healthy Faces, generated higher alpha, beta, and gamma activities in the right brain's occipital region—alpha [t(16) = 2.03; *p* < 0.05], beta [t(16) = 1.75; *p* < 0.05], and gamma [t(16) = 2.12; *p* < 0.05]—and higher beta activity in the temporal region [t(16) = 2.121; *p* < 0.05]. In the left brain, Recovered Faces exhibited increased gamma activity in the frontal [t(16) = 1.86; *p* < 0.05], parietal [t(16) = 1.75; *p* < 0.05], occipital [t(16) = 2.22; *p* < 0.05], and temporal [t(16) = 1.87; *p* < 0.05] regions.

## Discussion

This study explored the neural correlates of fear of COVID-19 contagion by stimulating participants with artificial-intelligence-generated faces of people presented as healthy, recovered from COVID-19 or infected by SARS-CoV-2. Globally, results documented a modulation of large functional brain networks as an effect of experimental manipulations, with selective recruitment of cortical (e.g., frontal lobes) and subcortical structures (e.g., amygdala) when participants observed COVID-19-related faces. These regions are part of the "social brain" network, a cortical-subcortical neural system that underlies social behaviour and emotion^19^. Consistent with fMRI results, EEG data showed distinct patterns of brain activity selectively associated with infected and recovered faces (i.e., delta and gamma rhythm). Taken together, EEG-fMRI results showed consistency among neuroimaging methods in detecting distinct brain states according to the different experimental conditions included in the study.

Within a broad bilateral fronto-temporo-parietal network, fMRI results showed that the involvement of frontal (e.g., superior and inferior frontal gyrus and orbitofrontal cortex), limbic (e.g., putamen and amygdala) and temporal-mesial (e.g., hippocampus) regions characterised higher-risk conditions exclusively (i.e., infected faces). These brain structures, on the one hand (i.e., subcortical limbic regions), may underlie the neural genesis of fear, and, on the other hand (i.e., frontal regions), are directly involved in its modulation. In particular, the orbitofrontal cortex, the superior and inferior frontal gyrus and the prefrontal cortex are key regions involved in top-down emotional control, the hippocampus is crucial for emotional memory, the amygdala is recruited in processing emotional events, and the putamen is involved in interpreting facial expressions^11,12,19,21,22,24–26^. Globally, this study’s fMRI results can be interpreted in light of the immunological characteristics of the AI-generated faces, which refer to their depicted COVID-19 infection status. These varying states (i.e., infected and recovered) likely elicit an enhanced, perhaps subconscious, perception of threat, particularly in the context of the pandemic and its associated risks. This framework of perceived threat due to the infection status of the faces can account for the observed fMRI outcomes. In addition, the fMRI analysis documented the engagement of bilateral ventral occipito-temporal brain structures (e.g., the inferior and medial temporal gyrus, lateral occipital cortex, parahippocampal gyrus) in all experimental conditions, thus irrespective of the faces' immunological status. Interestingly, this evidence is consistent with the literature on face processing, which underlines how such brain regions may be recruited in response to facial features (i.e., eyes, nose, and mouth) and are thought to play a role in both perceptual and affective aspects of face recognition^27–30^.

The fMRI results are coherently reflected in qualitative (eLORETA) and quantitative EEG analyses (i.e., spectral time–frequency analysis). Qualitatively, EEG data exposed extensive activations of bilateral fronto-temporo-parietal brain networks, which appeared modulated by experimental conditions. Specifically, infected and recovered faces shifted activations to central and frontal EEG regions, whereas healthy faces engaged occipital, temporal, and parietal regions. Quantitatively, EEG spectral analyses highlighted different brain rhythms as a function of experimental manipulations. Bilateral gamma activity was associated with viewing infected and recovered faces compared to healthy ones. Intriguingly, gamma activity may underline the activation of brain systems related to risk avoidance and fear extinction, as well as the social brain and amygdala response to fearful stimuli^31–35^. Also, gamma activity has been consistently linked with fear and anxiety^36^. Studies have shown increased gamma oscillations in the amygdala and prefrontal cortex during fear processing^37,38^. Along with gamma rhythm, infected and recovered faces produced stronger alpha oscillations in left occipital regions than healthy faces. In line with this finding, there is evidence in the literature about how exposure to negative affective stimuli, including fearful ones like infected faces, may elicit increased alpha activity in the occipital regions of the brain, which may counterintuitively reflect a reduction in the visual processing of these stimuli^39^. By contrasting infected with recovered faces, we found a stronger bilateral delta activity, which may suggest participants' attention to internal processing as a way to differentiate between similar categories of COVID-19-related stimuli^40^. This interpretation seems to be supported by the higher alpha activity we found for infected than recovered faces in the right occipital-parietal regions of the brain. Alpha oscillations in such regions are more sensitive to attentional modulation and visuospatial processing^41^. Finally, one may assume how differencing between recovered and healthy faces may require high-level cognitive processes, given the immunological proximity of such stimuli. The higher beta activity we found in temporal regions of the right brain may support such an interpretation as those areas, which include the right middle temporal gyrus we also found in the fMRI analyses, are more involved in face processing^30,42^. Congruently, beta oscillations may be more prominent in the right temporal regions during face perception tasks, reflecting the evaluation of facial features and their configuration^43–45^. Overall, the EEG activity modulation we found as the experimental manipulations change may signal the co-activation of distinct emotion- and cognition-related brain systems triggered by viewing COVID-19-related stimuli^15^.

As we introduced above, the general population experienced and continues to experience a significant psychological and social impact because of the COVID-19 pandemic^2–4^. Thus, while supporting the speculations proposed over the past few years in the COVID-19 literature, the results of this study underline how pandemic-related psychosocial effects may reverberate on the human social brain, thus influencing the most basic mechanisms of social interaction^15^. The primary limitation of this study lies in its small sample size, which resulted from the challenges in recruiting participants during the most critical phases of the COVID-19 pandemic in Italy. Future research in the area of pandemic-related risk perception should aim to at least double the sample size used in this study. However, despite the small sample size, the neural effects we report may signal a multiplicity of neural events that intervened during the COVID-19 pandemic. Consequently, one may assume such effects extend to any pandemic. Therefore, while this study aims to recount the past, it also aims to inform about possible future strategies to mitigate the psychosocial effects of pandemics, taking up Bill Gates' invitation to deal with the next pandemic^46^.

In conclusion, it should be noted that participants of this study activated fear-related neural mechanisms even when they were confronted with non-real faces, namely when they were alone, safe from COVID-19 infection, in an MRI scanner looking at computer-graphics-generated stimuli. Hence, one may easily understand how the weight of these mechanisms may dramatically impact social and cognitive functioning in real-life pandemic contexts^2–4^. This may explain the emergence of important clusters of psychopathologies during the pandemic^2^. Therefore, by detailing the impact of pandemic contexts on the human brain, this study's findings may have important implications for clinical interventions and emphasise the urgency of prompt interventions to prevent or reduce the extent of pandemic-related psychological discomfort and mental illness. Indeed, understanding the neurocognitive mechanisms of pandemic-related stress may help create public practices and policies to prevent its long-term effects, thus coping with what could be described today as *post-pandemic stress disorders*.

## Methods

### Participants

Participants were randomly recruited through advertisements posted on social media networks. Eighteen right-handed participants with self-reported normal vision were initially included in this study, which was conducted during the first waves of SARS-CoV-2 in Italy (i.e., October 2020–May 2021). One male participant was excluded from the sample due to the onset of a panic attack in the final minutes of the experiment. Therefore, the final number of participants in the study was seventeen (9 females; mean age = 26.35 years, SD = 3.9). The present study employed Python scripts based on *neurodesign* to determine the optimal design and sample size to increase the generalizability of the findings and enhance the study's reliability and validity^47^. All participants were Caucasians, had no history of neurological or psychiatric disorders, never contracted COVID-19 as of the study date, and gave informed consent to participate. All experimental procedures followed the ethical standards in the Declaration of Helsinki (1964). Accordingly, the study received approval from the local Ethics committee [i.e., *Istituto di Ricovero e Cura a Carattere Scientifico* Pascale (Naples, Italy), approval number: 06/2020].

### Materials

We used the same stimuli as devised by Federico and colleagues^15^, which consisted of eighteen images of faces (9 females; Fig. 1A) generated through machine learning by implementing a generative adversarial network^18^. The stimuli can be accessed online at https://osf.io/79xet/. We randomly divided the faces into three groups that corresponded to the three experimental conditions of the experiment: 6 × "Healthy Faces," 6 × "Infected Faces," and 6 × "Recovered Faces". Each experimental condition was matched for face sex (3 females for each condition). We changed the experimental condition assignment for each group of faces at every 6 participants. Therefore, each face was displayed in all the experimental conditions to control for possible effects generated by the specific emotional/perceptual salience of individual stimuli. Also, each facial expression was portrayed as emotionally neutral to counteract potential emotion-related biases. The stimuli were depicted in an MRI-compatible binocular visor with a resolution of 1024 × 768 px and a refresh rate of 60 Hz.

### Procedure

This study was conducted at the *Istituto di Ricovero e Cura a Carattere Scientifico* (IRCCS) Synlab SDN in Naples, Italy. Before the experiment commenced, written informed consent was obtained from each participant. Participants were then asked to self-report the absence of psychiatric or neurological diseases and confirm adequate visual acuity, right-handedness, and no prior COVID-19 diagnoses. Preparation for the EEG recording included EEG headset placement, after which participants were positioned in the MRI scanner. EEG and fMRI data were acquired simultaneously. Immediately after the participants were placed into the scanner, T1-weighted images were obtained, followed by the initiation of a functional protocol (refer to *MRI Data Acquisition* for details). A within-subject event-related experimental design was implemented. Experimental instructions were: "*Now, you will see some people's faces. Before each face, you will read whether the person is healthy, has recovered from COVID-19, or is currently infected by COVID-19. Please look at the faces as naturally as possible*". The experiment started with the administration of six stimuli for each experimental condition, resulting in the random presentation of eighteen faces as per the experimental visual flow (Fig. 1B). Consequently, each run consisted of 18 trials, distributed randomly among the three experimental conditions: 6 healthy faces, 6 infected faces, and 6 recovered faces. Each session lasted about 24 min. Following the EEG-fMRI paradigm, participants underwent a post-experimental interview to assess fear levels associated with potential real-life interactions with the stimuli's depicted individuals. Responses were collected on a 5-point Likert scale (1 = very little fear; 5 = very much fear). Results indicated that participants rated the faces of healthy individuals with a mean score of 1.23 (SD = 0.44) and recovered individuals with a mean score of 1.38 (SD = 0.65). In contrast, faces representing infected individuals received significantly higher average fear ratings, with a mean score of 4.22 (SD = 0.56). At the end of the experiment, participants were debriefed regarding its purposes.

### MRI data acquisition

Participants' structural and functional MR images were acquired using a Philips Achieva dStream 3T scanner and a 32-channel head coil. An *ad-hoc* acquisition protocol was implemented. Blood-Oxygen Level Dependent (BOLD) images were recorded with T2-weighted Echo-Planar Images (EPI) acquired with the multiband sequence. Functional images were collected as oblique-axial scans aligned with the anterior commissure–posterior commissure (AC–PC) line with the following parameters: 128 volumes per run, 45 slices, TR/TE = 2000/21.4 ms, flip angle = 90°, field of view = 240 × 240 mm^2^, slice thickness = 3 mm, voxel size = 3 × 3 × 3 mm^3^, multiband factor = 2. We acquired functional images continuously with no gaps between volumes. Therefore, the acquisition time (TA) is directly related to the repeat time (TR). Specifically, the TA with N = 45 slices is 1956 ms [TR − (TR/N)]. Structural T1-weighted images were collected using a 3D T1-TFE sequence (180 sagittal slices, TR/TE = 8.1/3.7 ms, flip angle = 8°, field of view 240 × 240 mm, slice thickness = 1 mm, voxel size = 1 × 1 × 1 mm^3^).

### MRI data pre-processing and denoising

MRI data were visually inspected by an experienced neuroradiologist (C.C.) for quality check. MRI data pre-processing and denoising were performed using the Functional Connectivity Toolbox (CONN; v. 21b; https://www.nitrc.org/projects/conn), as implemented in MATLAB (v. R2021b; https://mathworks.com/products/matlab.html). Pre-processing was carried out by implementing the standard CONN pre-processing pipeline, which included the following steps: (i) functional realignment and unwarp; (ii) slice-timing correction; (iii) outlier identification with CONN Artifact Detection Tools scrubbing; (iv) direct segmentation and normalisation in the Montreal Neurological Institute (MNI) reference space; (v) 8-mm full-width at half-maximum (FWHM) Gaussian smoothing. Then, pre-processed data were denoised using the CONN's default denoising pipeline, which included linear detrending, despiking and filtering (0.008 Hz < f < 0.09 Hz).

### Functional-connectivity MRI analysis

Task-dependent changes in functional connectivity (FC) were investigated using a generalized form of context-dependent psychophysiological interactions (gPPI) analysis with the CONN toolbox. gPPI is a variant of psychophysiological interactions analysis (PPI) designed to estimate effective FC across multiple experimental conditions. The gPPI-based general linear models comprise: (i) psychological predictors, namely the task effects convolved with a canonical hemodynamic response function; (ii) physiological predictors, which are the time series from the brain regions of interest (ROIs); and (iii) the interaction terms between psychological and physiological predictors^48^. Additionally, participants' gender was included as a covariate in the analysis to explore gender differences in social-emotional processing^49^. Therefore, a hypothesis-driven gPPI analysis was performed to identify task-modulated changes in FC patterns covarying with the experimental conditions in the context of this study's event-related experimental design. For each trial, the psychological predictors were modelled by considering the appearance on screen of the face stimulus from its onset to its end (see the experimental visual flow in Fig. 1B). In this way, the FC analysis was restricted to the participants' visual encoding of the face stimuli. Given the exploratory nature of this study, all the cortical and subcortical areas of the human brain were selected as ROIs from the CONN's default cortical atlas, i.e., the Harvard–Oxford atlas as distributed with FSL (https://fsl.fmrib.ox.ac.uk).

### EEG data acquisition

EEG signals were recorded using BrainVision Recorder (Brain Products, Munich, Germany) and MR-compatible AC amplifiers (BrainAmp MRplus; Brain Products, Munich, Germany) during the MRI scanning process. The EEG electrode cap (BrainCapMR 96, Brain Products, Munich, Germany) was equipped with 95 active sintered silver/silver chloride electrodes arranged according to a modified 10/10 system. FCz electrode served as the reference, while the AFz electrode acted as the ground. An ECG electrode was also used to capture pulse-related artefacts for subsequent correction. The ribbon cable connecting the electrode wires and amplifiers was secured with sandbags on foam cushions to minimise interference from the scanner's vibrations. Electrode skin impedance was maintained below 10kΩ. The EEG data were sampled at 5000 Hz, with an amplitude resolution of 0.5 μV.

### EEG data pre-processing

Brain Vision Analyzer 2.1 (Brain Products, Munich, Germany) was used for offline EEG analysis. The continuous magnetic resonance artefact was corrected by generating an *ad-hoc* sliding average artefact template. The dataset was resampled to a sampling rate of 256 Hz to facilitate further analysis. A low pass filter with a cut-off frequency of 70 Hz, a high pass filter with a cut-off frequency of 0.5 Hz, and a Notch Filter were applied for signal filtering. The cardioballistic artefact was corrected by employing a semi-automatic pulse template on the ECG channel and subtracting the pulse artefact from all channels. Non-stereotyped artefacts were removed through visual inspection, including movements and channel drifts. Independent Component Analysis was then employed to identify and eliminate eye movements, residual gradient artefacts, and other artefacts that were present. The remaining data epochs were visually inspected and rejected if deemed artificial or noisy. The EEG data were re-referenced to a common average. The EEG data were segmented around stimuli markers from − 500 to 1500 ms and exported for time–frequency spectral and source analysis.

### EEG time–frequency spectral analysis

The EEG time–frequency spectral analysis was conducted using Brainstorm^50^. A new Brainstorm protocol was created using the default "MNI-ICBM152" brain-anatomy template. The scalp surface was automatically calculated and then registered with the coordinates of the EEG cap. All EEG data were segmented into epochs and averaged across the three experimental conditions, spanning from − 500 to 1500 ms. Delta, alpha, beta, and gamma activities were analysed using ad-hoc time–frequency Morlet-Wavelet analyses^51^. The frequency values of interest were extracted by grouping EEG channels into five regions for both hemispheres: frontal (Fp1, Fz, F1, F3, F5, F7, AF3, AF7, F9, AFF1h, FFC5h, FFC1h, Fp2, F2, F4, F6, F8, AF4, AF8, F10, AFF2h, FFC2h, FFC6H); central (C1, C3, CCP1h, FCC3h, CP1, CP3, FC1, FC3, CZ, CCP5h, CPz, C2, C4, CPP2h, FCC4h, CP2, CP4, FC4, FC2, CPP6h); parietal (P1, P3, P5, P7, P9, Pz, TPP7h, CPP3h, CPz, PPO9h, P2, P4, P6, P8, P10, TPP8h, CPP4h, PPO10h); occipital (PO9, Ol1h, O1, O9, PPO1h, PO3, PO7, POz, OZ, PO10,Ol2h, O2, O10, PPO2h, PO4, PO8); temporal (FT9, FT7, FTT7h, T7, C5, FC5, CP5, TP7, TP9, FT10, FT8, FTT8h, T8, C6, FC6, CP6, TP8, TP10). The EEG spectral analysis considered the macroscopic co-activation pattern derived from fMRI analyses, which offers higher spatial resolution than EEG. Thus, multiple fMRI-driven one-tailed paired t-tests were conducted to examine the differences in EEG oscillations. These tests focused on the delta, theta, alpha, beta, and gamma activity within the frontal, central, parietal, occipital, and temporal EEG regions of the left and right brain hemispheres. The tests were performed to evaluate the following comparisons among experimental conditions: (i) Infected Faces > Healthy Faces; (ii) Infected Faces > Recovered Faces; (iii) Recovered Faces > Healthy Faces.

#### EEG source-activation qualitative analysis

The EEG source analysis used the exact low-resolution electromagnetic tomography (eLORETA)^23,52^. To estimate cortical source activation from scalp electrodes, a 3-shell sphere head model was computed. This model was based on the Montreal Neurological Institute cerebral-shape template, co-registered with the Talairach brain atlas. For the source analysis, a frequency resolution of 0.5 Hz was utilised. This resolution was achieved using artefact-free EEG epochs that spanned 2 s (− 500 to 1500 ms). The input for eLORETA consisted of a set of artefact-free EEG epochs recorded from the 95 scalp electrodes, positioned according to the 10–10 montage scheme. The eLORETA source estimation generated a collection of estimations of neural ionic current densities in the cortical grey matter. This collection comprised 6239 voxels with a resolution of 5 mm, where each voxel represented an analogous current dipole. The Talairach brain atlas coordinates provided the corresponding Brodmann area (BA) for each voxel. A regional analysis was conducted by grouping the 95 scalp electrodes into six regions of interest (ROIs): frontal (BA 8, 9, 10, 11, 44, 45, 46, 47); central 1 (BA 1, 2, 3, 4, 6); parietal (BA 5, 7, 30, 39, 40, 43); occipital (BA 17, 18, 19); temporal (BA 20, 21, 22, 37, 38, 41, 42); central 2 (BA 31, 32, 33, 34, 35, 36). For each ROI, the average of the normalised eLORETA current density values, computed across all single voxels within that ROI, served as the eLORETA solution for the respective macro-lobar ROI. To analyse the cortical sources in each experimental condition and across different frequency bands and macro-regions, the solutions from the six ROIs of eLORETA were averaged. This allowed for qualitative comparisons of source activations as the experimental conditions varied.

#### Statistical analyses

Statistics of the MRI functional connectivity analyses (FC) were assessed with the standard toolbox included in CONN. Quantitative EEG data were instead analysed with R (v. 4.2; https://www.r-project.org). Analyses included an alpha level of 0.05, with False Discovery Rate (FDR) correction for fMRI data related to ROI-to-ROI multiple comparisons^53^. The FDR correction was chosen due to the exploratory nature of the study. Indeed, FDR offers a balance between identifying significant findings and controlling for false positives. This approach is less conservative but more sensitive in detecting true effects, which is crucial in exploratory studies where the priority is to uncover new patterns^54^.

## Data Availability

The data that support the findings of the present study will be available from the corresponding author upon reasonable request.
